# Supplementary material for: Ground-truth validation of uni- and multivariate lesion inference approaches
Source: Brain Commun. 2024 Jul 26;6(5):fcae251. doi: 10.1093/braincomms/fcae251 (PMC11406464; doi:10.1093/braincomms/fcae251)
Supplement: fcae251_Supplementary_Data [file fcae251_supplementary_data.docx]

**Supplementary material**

**Displacement in LSM**

In order to assess the source of the error introduced by univariate approaches, specifically LSM, we quantified the displacement from the true location by computing the total mis-inferences for every ground-truth lesion model across all the inferred BAs, both for graded and median threshold lesion models, and correlated it with (1) the median of relative lesion size per area across all cases (rho = 0.33 p = 0.04 for the graded lesion model and rho = 0.32 p = 0.04 for median threshold lesion model; scatter plot in Supplementary Figure 1A and 1B respectively); (2) the median of absolute lesion size per area across all cases (rho = 0.63 p < 0.001 for the graded lesion model and rho = 0.58 p < 0.001 for median threshold lesion model, scatter plot in Supplementary Figure 2A and 2B respectively) and (3) the absolute size per area (rho = 0.27 p = 0.09 for the graded lesion model and rho = 0.23 p = 0.15 for median threshold lesion model, scatter plot in Supplementary Figure 3A and 3B respectively). The results suggested that it is the absolute lesion size of regions that matters and not the size of the regions as such.

**Double-regions simulations – Low vs high correlations pairs**

Among the double-region models we performed simulations randomly selecting 50 pairs of regions within a group of regions having high correlations of lesion size and a group with low correlation of lesion size. The results in term of accuracy, sensitivity, specificity and mis-inference error, both for binary and graded lesion models, assuming both synergistic and redundant operations, are shown in Supplementary Table 1. The results suggest that the mis-inference of the univariate methods does not seem to depend very strongly on whether the two areas have similar lesion patterns.

| **Inference method** | **Accuracy %** | **Accuracy_weighted %** | | **Sensitivity %** | | **Specificity %** | **Misinference error %** |
| --- | --- | --- | --- | --- | --- | --- | --- |
| **Double source binary synergistic - LC** | | | | | | | |
| LSC | 22 | | 22 | | 100 | 18 | 90 |
| LSM | 33 | | 33 | | 100 | 30 | 86 |
| MAPP | 100 | | 100 | | 100 | 100 | 0 |
| MSA | 100 | | 100 | | 100 | 100 | 0 |
| **Double source binary synergistic - HC** | | | | | | | |
| LSC | 29 | | 29 | | 100 | 26 | 87 |
| LSM | 43 | | 43 | | 100 | 40 | 83 |
| MAPP | 99 | | 99 | | 100 | 99 | 0 |
| MSA | 100 | | 100 | | 100 | 99 | 1 |
| **Double source binary redundant - LC** | | | | | | | |
| LSC | 35 | | 35 | | 100 | 32 | 90 |
| LSM | 51 | | 51 | | 98 | 49 | 87 |
| MAPP | 93 | | 94 | | 88 | 94 | 8 |
| MSA | 95 | | 95 | | 91 | 95 | 15 |
| **Double source binary redundant - HC** | | | | | | | |
| LSC | 30 | | 30 | | 100 | 27 | 88 |
| LSM | 45 | | 45 | | 100 | 42 | 83 |
| MAPP | 99 | | 99 | | 99 | 99 | 0 |
| MSA | 100 | | 100 | | 100 | 100 | 0 |
| **Double source binary mutual inhibition - LC** | | | | | | | |
| LSC | 27 | | 27 | | 100 | 24 | 87 |
| LSM | 39 | | 39 | | 99 | 36 | 86 |
| MAPP | 97 | | 97 | | 100 | 97 | 1 |
| MSA | 98 | | 98 | | 100 | 97 | 1 |
| **Double source binary mutual inhibition - HC** | | | | | | | |
| LSC | 55 | | 55 | | 95 | 53 | 81 |
| LSM | 72 | | 71 | | 84 | 71 | 78 |
| MAPP | 87 | | 87 | | 100 | 87 | 2 |
| MSA | 90 | | 90 | | 100 | 90 | 2 |
| **Double source graded synergistic - LC** | | | | | | | |
| LSC | 19 | | 19 | | 100 | 15 | 89 |
| LSM | 33 | | 33 | | 100 | 30 | 87 |
| MAPP | 54 | | 54 | | 100 | 52 | 7 |
| MSA | 63 | | 63 | | 100 | 61 | 18 |
| **Double source graded synergistic - HC** | | | | | | | |
| LSC | 26 | | 26 | | 100 | 22 | 86 |
| LSM | 39 | | 39 | | 100 | 36 | 82 |
| MAPP | 41 | | 41 | | 99 | 38 | 10 |
| MSA | 52 | | 52 | | 100 | 50 | 14 |
| **Double source graded redundant - LC** | | | | | | | |
| LSC | 19 | | 19 | | 100 | 15 | 89 |
| LSM | 34 | | 33 | | 100 | 30 | 87 |
| MAPP | 59 | | 59 | | 100 | 57 | 8 |
| MSA | 64 | | 64 | | 100 | 63 | 20 |
| **Double source graded redundant - HC** | | | | | | | |
| LSC | 26 | | 26 | | 100 | 22 | 86 |
| LSM | 39 | | 39 | | 100 | 36 | 82 |
| MAPP | 43 | | 43 | | 100 | 40 | 11 |
| MSA | 53 | | 53 | | 100 | 51 | 15 |
| **Double source graded mutual inhibition - LC** | | | | | | | |
| LSC | 22 | | 22 | | 100 | 18 | 90 |
| LSM | 41 | | 41 | | 88 | 39 | 90 |
| MAPP | 86 | | 86 | | 72 | 87 | 14 |
| MSA | 94 | | 94 | | 80 | 95 | 19 |
| **Double source graded mutual inhibition - HC** | | | | | | | |
| LSC | 30 | | 30 | | 100 | 26 | 87 |
| LSM | 53 | | 52 | | 100 | 50 | 84 |
| MAPP | 74 | | 74 | | 57 | 75 | 35 |
| MSA | 91 | | 91 | | 92 | 91 | 19 |

**Supplementary Table 1: LC**: Low correlation pairs of regions, **HC**: High correlation pairs of regions. **TP**: true positive; **TN**: true negative; **FP**: false positive; **FN**: false negative. **Accuracy** = *(TP+TN)/(TP+TN+FP+FN)*; **Accuracy_weighted** = *(TP*(1-var(diag))+TN*(1-sum(off_diag)))/(TP+TN+FP+FN)*. **Sensitivity** = *TP/(TP+FN)*; **Specificity** = *TN/(TN+FP)*; **Mis-inference error** = sum of all inferred regional contributions beyond the ones of the target/s. All the quantities are computed on the normalized significant values in the matrices. For all the simulations we used a regression tree predictor (default parameters).

**Supplementary Figure 1A**

**Supplementary Figure 1B**

**Supplementary Figure 2A**

**Supplementary Figure 2B**

**Supplementary Figure 3A**

 **Supplementary Figure 3B**

| **Inference method** | **Accuracy %** | **Accuracy_weighted %** | **Sensitivity %** | **Specificity %** | **Misinference error %** |
| --- | --- | --- | --- | --- | --- |
| **Double source binary synergy with 10% noise** | | | | | |
| LSC | 83 | 83 | 100 | 82 | 89 |
| LSM | 81 | 80 | 100 | 80 | 85 |
| MAPP | 100 | 100 | 100 | 100 | 18 |
| MSA | 98 | 98 | 100 | 98 | 36 |
| **Triple source binary redundant** | | | | | |
| LSC | 32 | 32 | 97 | 27 | 89 |
| LSM | 45 | 45 | 94 | 41 | 85 |
| MAPP | 89 | 89 | 78 | 90 | 12 |
| MSA | 91 | 91 | 81 | 91 | 26 |
| **Triple source graded redundant** | | | | | |
| LSC | 23 | 23 | 100 | 17 | 86 |
| LSM | 31 | 31 | 100 | 26 | 86 |
| MAPP | 37 | 37 | 100 | 32 | 14 |
| MSA | 44 | 44 | 100 | 40 | 25 |

**Supplementary Table 2: Additional simulations with noise and with three regions responsible for a putative functional score.** Double source binary synergy when the intact score (1) is changed to an impaired one (0) in 10% of the cases. **TP**: true positive; **TN**: true negative; **FP**: false positive; **FN**: false negative. **Accuracy** = *(TP+TN)/(TP+TN+FP+FN)*; **Accuracy_weighted** = *(TP*(1-var(diag))+TN*(1-sum(off_diag)))/(TP+TN+FP+FN)*. **Sensitivity** = *TP/(TP+FN)*; **Specificity** = *TN/(TN+FP)*; **Mis-inference error** = sum of all inferred regional contributions beyond the ones of the target/s. All the quantities are computed on the normalized significant values in the matrices.

| **models** | **mean ± std / range** | | | |
| --- | --- | --- | --- | --- |
|  | *LSM (t-score)* | *LSC (correlation coefficient)* | *MAPP (prediction error contribution)* | *MSA (normalized contribution)* |
| single source binary | 4.82 ± 5.43  range (-4.21; 24.08) | 0.21 ± 0.2  range (-0.25; 0.84) | 0.0041 ± 0.026  range (0; 0.32) | 0.024 ± 0.0038  range (0; 1) |
| single source binary + 10% noise | 3.04 ± 3.59  range (0; 21.44) | 0.15 ± 0.14  range (-0.13; 0.89) | 0.0013 ± 0.0016  range (-0.03; 0.26) | 0.019 ± 0.1  range (-0.05; 1) |
| double source binary synergy | 5.91 ± 5.09  range (-4.45; 24.08) | 0.27 ± 0.2  range (-0.23; 0.84) | 0.0072 ± 0.033  range (-0.001; 0.31) | 0.024 ± 0.1  range (0; 0.65) |
| double source binary synergy + 10% noise | 4.19 ± 3.90  range (-4.13; 20.27) | 0.2 ± 0.15  range (-0.17; 0.72) | 0.0016 ± 0.0007  range (-0.002; 0.08) | 0.018 ± 0.06  range (-0.05; 0.34) |
| double source binary redundant | 4.66 ± 4.37  range (0; 24.08) | 0.18 ± 0.15  range (-0.15; 0.76) | 0.0032 ± 0.016  range (-0.01; 0.24) | 0.024 ± 0.1  range (-0.11; 1) |
| double source binary redundant + 10% noise | 4.34 ± 4.14  range (0; 22.47) | 0.19 ± 0.14  range (-0.15; 0.7) | -0.036 ± 0.012  range (-0.08; 0.14) | 0.019 ± 0.09  range (-0.34; 1) |
| double source binary redundant + 50% noise | 3.26 ± 3.33  range (0; 16.85) | 0.143 ± 0.11  range (-0.11; 0.51) | -0.053 ± 0.005  range (-0.1; 0.04) | 0.0086 ± 0.065  range (-0.43; 0.52) |
| clinical data (Zavaglia et al. 2015) | range: (0.8; 4.4) | range: (-0.06; 0.52) | range: (-0.09; 0.5) | range: (-0.09; 0.28) |

**Supplementary Table 3: Effect size of target variables in different set up and noise levels for all four approaches and for one clinical dataset.** Specifically, we report the t-scores for LSM, the correlation coefficient for the LSC, the prediction error contribution for MAPP and the normalized contribution for MSA. All results are reported as mean ± std and the range of values, except for the clinical data where the range is reported.
